# Diversity in Autistic Play: Autistic Adults' Experiences

**DOI:** 10.1089/aut.2023.0008

**Published:** 2024-06-17

**Authors:** Emma Pritchard-Rowe, Carmen de Lemos, Katie Howard, Jenny Gibson

**Affiliations:** ^1^ Play & Communication Lab, Play in Education, Development and Learning (PEDAL) Centre, Faculty of Education, University of Cambridge, Cambridge, United Kingdom.; ^2^ School of Education, University of Exeter, Exeter, United Kingdom.

**Keywords:** autism, play, autistic perspectives, neurodiversity

## Abstract

**Background:**

Play is important for mental health and well-being. Descriptions of autistic play have typically focused on “deficits” and are based on comparisons to neurotypical “norms”. According to the neurodiversity paradigm, it is important that autistic voices are highlighted and that difficulties, differences, and strengths are explored. With this in mind, we designed the present study to focus on the experiences and perspectives of autistic people concerning the topic of autistic play.

**Methods:**

We conducted a consultation with autistic stakeholders, as well as with parents and teachers of autistic individuals to help us design the study and interview questions. We used semi-structured interviews with 22 autistic adults aged 18–57 years (clinically confirmed diagnosis, *n* = 21; self-diagnosed, *n* = 1) who live in the United Kingdom. We analyzed the data using interpretative phenomenological analysis to identify themes.

**Results:**

We found important commonalities and differences in the ways that socialization in play, imaginary play, and flow (a state involving intense focus on the play) are experienced. Autistic adults discussed the importance of both solitary play and social play, with solitary play having an important recuperative function. They also reported preferences for parallel play and playing with similar autistic people. They also discussed imaginary play experiences, including social role-play and grounded-in-reality play, and the dual nature of flow experiences during play.

**Conclusions:**

The findings of this study contrast with deficit-focused understandings of autistic play and build on neurodiversity-informed studies. We highlight, for example, the importance of considering the different circumstances under which solitary play or social play are preferred, as well as the importance of taking an individual approach to play. We encourage wider understanding and acceptance of these play preferences and experiences to support autistic people's well-being.

## Community brief

Why is this an important issue?

Usually, descriptions of autistic play are negative and are based on what non-autistic people think autistic play should be like. We think that it is important to challenge this by exploring autistic play in a more balanced way and focusing on what autistic people say about their play.

What was the purpose of this study?

We wanted to find out how autistic adults experience play. We were also interested in how they think their play is different from non-autistic play.

What did the researchers do?

We asked autistic and non-autistic people to help us design our study and interview questions. We interviewed 22 autistic adults and asked them about their play now or when they were younger. We then analyzed the data using a qualitative method called interpretative phenomenological analysis. This meant that we could identify themes covering the group's views and experiences.

What were the results of the study?

We found a range of play experiences and different ways in which some autistic adults play. Many autistic adults talked about the importance of both playing by themselves and with others, with solitary play described as being recuperating. Some described preferring play that involves playing nearby other people without necessarily interacting with them directly. Many preferred to play with similar autistic people. Several autistic adults discussed engaging in imaginary play activities such as role-play and preferring play that is based on reality. They also described the benefits and limitations of something called a flow state, which involves intense focus on the play.

What do these findings add to what was already known?

Our findings highlight how autistic adults like to play and different ways some autistic adults experience play. For example, we highlight the situations where solitary play and social play are preferred, and the importance of understanding that different people like to play in different ways.

What are potential weaknesses in the study?

We did not involve autistic people beyond consultation. We think that a better understanding of autistic play can come from using approaches with greater autistic involvement, such as co-production. Most of our participants were White, highly educated, and primarily communicate using speech. We do not know if other autistic people would experience play in a similar way.

How will these findings help autistic adults now or in the future?

Understanding and acceptance of the ways in which autistic people like to play can help support autistic people's well-being. In practice, this involves limiting the use of supports that aim to make autistic people's play “normal” and instead encouraging authentic autistic play.

## Background

Play is important for nurturing aspects of linguistic, social, cognitive, and emotional development^1^ and is important for well-being and mental health.^2,3^ Play is notoriously difficult to define, and no agreement exists about how to define it.^4^ However, it is often characterized as behavior that is fun or rewarding, voluntary, and intrinsically motivated (done for its own sake).^5,6^ Two key types of play are solitary play and social play. Traditionally, social play has been theorized within the developmental psychology literature as a behavior that reflects social competence.^7^ Defined as play with at least one other person, social play is often contrasted to solitary play (playing by oneself), and different categories of social play have been proposed, which reflect increasing social interaction and competence.^8,9^ For example, this ranges from parallel play, involving playing in close proximity to others but not interacting with them, to cooperative or organized supplementary play, involving goal-oriented, playful interaction with others.^9^

Typically, how autistic play has been described or understood has been deficit-focused. For instance, autistic play has been characterized by “deficits” in imaginary play^10^ and social play.^11,12^ Descriptions have also tended to frame play behaviors or preferences in a negative way, for example, in relation to stimming^13^ and preferences for solitary play. Autistic “deficits,” including play “deficits,” tend to be interpreted as such based on non-autistic interpretation of observable autistic play behaviors, often by comparing these behaviors with neurotypical “norms”.^14–17^ As well as focusing on “deficits” then, understandings of autistic play have tended to neglect autistic perspectives. These autistic play “deficits” are reflected in diagnostic criteria and assessment practices,^18^ where they are pathologized and viewed as behaviors that need to be “fixed” or normalized. Aiming to counter this deficit focus, this article reports a study that aimed to explore autistic play from a neurodiversity-informed perspective.

In line with understanding autism from a neurodiversity-informed perspective, autistic play can be conceptualized as a phenomenon characterized by difficulties, differences, and strengths.^15,19–23^ For example, our preliminary consultation indicated that some autistic people have difficulties accessing types of play most appropriate to their needs and preferences. Accordingly, one aspect of exploring autistic play from a neurodiversity-informed perspective involves understanding autistic play in a balanced way and using more neutral or positive language. This is important as advocates of the neurodiversity perspective argue that the reliance of many professionals and researchers on deficit-focused language can be seen as a form of ableism that is associated with negative outcomes for autistic people, such as exacerbating stigma and marginalization.^24^ Furthermore, the focus on the “deficits” and increased stigma can contribute to masking and poorer mental health and well-being for autistic people.^25^

Another important aspect of adopting a neurodiversity-informed perspective to understanding autistic play is to center autistic voices in research. There is limited published research on experiences of autistic play involving autistic people in decision-making around what (and how) the research gets done (often termed “participatory research”), which is important in making research more relevant to autistic people and meaningfully improving their lives.^26–28^ Furthermore, as understandings of autistic play itself have tended to be based on non-autistic views, understanding autistic perspectives of play can improve our understanding of this phenomenon.

Despite the importance of understanding autistic play from a neurodiversity-informed perspective, there is a relative lack of research that focuses on this. Moving away from the deficit focus, Conn^29^ analyzed autistic autobiographies to identify patterns of enjoyable childhood play experiences. Conn identified several features of an autistic play culture (shared play features or experiences) including preferences for sensory play, imaginary play that is based on real life, and certain ways of interacting with others. Conn and Drew^30^ explored the views of non-autistic siblings on childhood play they enjoyed with their autistic siblings, with enjoyable sibling play including imaginary, construction, and physical play.

Focusing on outdoor play, Fahy et al.^31^ explored autistic children's preferences using a multimethod approach that included interviews with children. The autistic children preferred many play forms, including active play with different sensory inputs, risky play, imaginative play, and social play. Children were also observed to experience a flow state, a subjective experience characterized by intense involvement or concentration in an activity.^32^ Most recently, Pavlopoulou et al.^33^ interviewed autistic adolescents about their motivations and the benefits of playing online video games. Example findings include that while immersion within a game (i.e., a flow state) can have a negative impact on daily routines, gaming promotes a sense of agency and belonging and supports emotional regulation.

Overall, such research highlights a variety of positive or enjoyable autistic play experiences, as well as preferences and motivations for autistic children and adolescents. This research also demonstrates the importance of autistic play for positive mental health and well-being, as Pavlopoulou et al.'s^33^ research directly demonstrates the benefits of gaming for autistic people's well-being, and by definition, enjoyable autistic play is inherently related to well-being. In addition, although flow can have a negative impact, it has also been linked to autistic people's well-being.^34^ Given that autistic people commonly experience mental health conditions,^35,36^ this reinforces the importance of shifting away from the deficit focus. Instead, research should develop more balanced understandings of autistic play that have the potential to better support autistic well-being.

The research outlined also included play types that have previously been viewed as autistic “deficits,” such as imaginary play or social play. This further supports the need to shift away from the deficit focus, and explore autistic perspectives, as these can reframe how we understand autistic play. Nonetheless, exploring autistic play from a neurodiversity-informed perspective remains an underdeveloped area of research. In this article, we aim to learn more about autistic adults' perspectives on this topic, including how autistic play is different from non-autistic play.

### The present study

To counter the deficit focus and understand autistic play from a neurodiversity-informed perspective, we conducted a study focusing on autistic perspectives of play. This project sought to examine autistic experiences of play and play-based assessments administered by professionals (https://osf.io/e8uvh). In this article, we focus on autistic experiences of play. Accordingly, we aimed to explore the experiences and meaning of play from an autistic perspective, identifying common themes linked to the process, activities, and motivations involved in the phenomena of autistic play, and to identify any perceived differences of autistic play to non-autistic play from an autistic perspective. This study involved autistic adults who reflected on both their contemporary experiences of play and their experiences of play during childhood. We operationalized a neurodiversity-informed approach by adopting a methodological approach that centers autistic perspectives and adopting a balanced rather than deficit-focused lens when interpreting the data.

The specific research questions are as follows:

1. How do autistic individuals construct play experiences?
2. What do autistic individuals consider important about the ways in which they engage in play?
3. Do autistic individuals identify any differences between autistic and non-autistic play?

## Methods

### Ethical considerations

Ethical approval was awarded by the Faculty of Education Research Ethics Committee. Participants provided informed written and verbal consent before completing interviews. We use pseudonyms in this article to protect participants' anonymity.

### Community involvement in study design

Autistic adults, parents of autistic people, and professionals working with autistic people took part in a wider consultation process, with a subset selected to help us design the study and interview questions. See https://osf.io/92gud/ for more details.

### Methodological framework

We adopted a qualitative design using interpretative phenomenological analysis (IPA).^37,38^ We adopted this method as its interpretative phenomenological epistemology permits an understanding of individuals' lived experiences of a particular phenomenon, in line with the aims of the project. Importantly, IPA is appropriate because it views the individual as an expert of their own experiences, thus aiming to reduce power imbalances,^39,40^ and because of the increased researcher reflexivity that is associated with the “double hermeneutic”, in which the researcher seeks to make sense of the participant's sense making of their own experiences.^37,38^ This is particularly pertinent given the double empathy problem,^41^ which acknowledges that non-autistic researchers may struggle to understand autistic experiences.

### Participants

We recruited a purposive sample of 22 autistic adults from the United Kingdom aged 18–57 years (*M* = 39.00, standard deviation = 11.39). We recruited participants through advertising on social media, contact with autistic and non-autistic stakeholders who took part in the previously discussed consultation, and advertising via an autism research charity. We included participants if they had co-occurring conditions. See Table 1 for participant demographic information.

**Table 1.** Participant Demographic Information

Demographic	*n*	%
Gender		
Male	10	45
Female	10	45
Nonbinary/agender	2	9
Ethnicity		
White	20	91
Mixed/multiple ethnic groups	1	5
Other ethnic group	1	5
Highest level of education		
Primary school	1	5
High school	2	9
Further education college	3	14
Undergraduate degree	10	45
Postgraduate degree	5	23
Other	1	5
Diagnosis type		
Professional diagnosis	21	95
Self-diagnosed	1	5

Percentages may not total 100 due to rounding.

### Procedure

We conducted semi-structured interviews with participants to explore the research aims and questions. The interviews adopted either a written (email, Skype instant messenger) or verbal (phone, Zoom, Skype) format depending on each participant's preference. We recorded the audio interviews.

The interviews focused on three areas: experiences of play, comparisons between autistic and non-autistic play, and experiences of assessment and supports using play (Supplementary Material 1 or see https://osf.io/92gud/ for interview schedule). We encouraged the participants to think about their play now and/or when they were younger. We transcribed the audio interviews verbatim using NVivo 12 software and anonymized all transcripts.

### Data analysis

The first author led the analysis, with contributions from the other authors. The process was informed by IPA “guidelines”^37,38^ and discussions with team members. We conducted the analysis using various software (NVivo 12, Miro, Excel). An iterative process was undertaken to analyze the data (Table 2), resulting in the master list of superordinate and subordinate themes.

**Table 2.** Interpretative Phenomenological Analysis Process Adopted in This Study

Step	Description
1. Initial familiarization and noting	Involved reading and re-reading (plus listening to the recording) of the transcript. Initial notes were made relating to three types of exploratory comments: descriptive, linguistic, and conceptual.
2. Develop emergent themes	Concise phrases were developed that summarize various exploratory comments.
3. Repeat steps for all transcripts	Steps 1–2 were repeated for each transcript.
4. Identify superordinate and subordinate themes for master list	Following guidelines with larger samples,^37,38^ once each transcript was analyzed, the focus shifted to analyzing patterns across the transcripts. This included identifying recurrent themes–themes present in at least 50% of the transcripts. An iterative process of categorization and reduction was undertaken to create the master list of superordinate and subordinate themes. This involved identifying the most recurrent and “important” superordinate themes.

We identified the most recurrent and “important” superordinate themes based on relevance to the research questions and to the wider context of the study. The approach adopted aimed to capture a balance between commonality and variation in autistic people's experiences, in line with IPA's idiographic commitment.^37,38^

We took steps to enhance the trustworthiness^42^ of the analysis. During the initial analysis stage, two members of the research team independently analyzed one transcript and identified similar themes. Additionally, ongoing discussions and revisions of the master list of themes took place based on the analysis conducted. Aiding transparency and reflexivity, the first and second authors kept a reflective diary and the authors have reflected on their positionality (Supplementary Material 2).

## Findings

Table 3 shows the superordinate and subordinate themes, and the prevalence of the superordinate themes, following Smith et al.'s^37,38^ recommendations. We discuss these themes and illustrate them with supporting quotations. We selected exemplar quotations representing a range of different participants.

**Table 3.** Overview of Themes

Superordinate theme	Subordinate theme	Prevalence
1. Socialization in play	1.1. Solitary play versus social play	22
	1.2. Preferences in social play	
2. Imaginary play	2.1. Engaging in imaginary play	20
	2.2. Grounded in reality	
3. Flow	3.1. Experiences of flow	16
	3.2. Benefits and limitations	

### 1. Socialization in play

There were a variety of experiences relating to solitary play and social play. We captured these under two subordinate themes. We used these traditional categorizations of play as a useful starting point for understanding the autistic play experiences described by participants in this study. However, we acknowledge that the term “solitary play” may have positive connotations for some and connote marginalization or exclusion for others.

#### 1.1. Solitary play versus social play

For most participants, their play was generally more solitary than social, or they had a preference for solitary play. For example, Brianna stated that they are “probably more inclined to solitary play”. A few participants reflected that this preference might represent a difference between autistic and non-autistic play: “ours are normally more solitary” (Peter). This did not mean that participants did not engage in social play; many valued both solitary play and social play and talked about the different purposes of these types of play. The main benefit of social play, unsurprisingly, was the various social benefits it offered. These benefits included the opportunity to socialize, bond, or connect with others and to develop or practice social skills. For example, Amanda pointed out that “it's necessary to play with others, because that's how you learn about socialising, and taking turns and letting other people take the lead sometimes if you can, and it's how you learn kind of to communicate socially”.

In contrast, for many, solitary play serves a recuperating or relaxing function. For example, Lauren argued that “if I just wanted my own chill time or wanted to just be on my own and chill and play, then I'd want solitary play”. Some participants articulated that they preferred solitary play if they were tired or if their resources were drained in some way. This might be due to the demands of social play, as highlighted by Jason: “I loved it cos it was exciting and imaginative and I was socially interacting with a friend who I really liked. But yeah it was exhausting and I couldn't do it for very long”.

This notion of social play as both rewarding and draining is eloquently summarized by Nathan, who described it as a “double-edged sword”:

“When I'm feeling overwhelmed what I need at that moment is something repetitive and comforting, so at that time I need solitary play. Or if my mind is sort of very active with rumination and that kind of thing then I need something to take my mind off it, and so solitary play serves that purpose. But when I'm feeling more resourceful and have some energy to spare, then I definitely would prefer social play. I think that's very valuable to me, just as way of not feeling lonely really and [it] gives me a sense of self-worth … so I think, I definitely enjoy social play more but I need to be in the right frame of mind to be able to tolerate it, and definitely afterwards I'm very drained and often I will ruminate afterwards about how I came across and that kind of thing. So it's a bit of a double-edged sword.”

Supporting earlier findings, it is also clear from Nathan's account, specifically the use of phrases such as “take my mind off”, that solitary play is important for recuperating. Overall then, for some participants, their preference for one type of play over the other depends on their available resources or their needs at a specific moment.

#### 1.2. Preferences in social play

Some participants described potential differences in social play between autistic and non-autistic people. A few participants described autistic social play as play that takes place in close proximity to others but without requirement for interaction or collaboration. This is akin to parallel play (e.g., Parten).^9^ For example, Fiona commented:

“What I considered social play isn't necessarily what other children would. So, for me social play is ‘I'm going to do my drawing here and you're gunna sit next to me and do your drawing next to me and we might have a chat and look at each other's drawings’ … to me that is social because you're together, you're doing an activity.”

Here, Fiona demonstrates her awareness of how her definition of social play is different from other people's. Similarly, Robert articulated that “social play for autistic people … was actually just being with the group of people and interacting with them from time to time, but actually, almost kind of doing your own thing and being on the side-lines”.

A few participants described their tendency or preference toward playing in smaller groups. Jason “really struggled to enjoy play [sic] with more than one person”. One participant reflected that this preference represents a difference between autistic and non-autistic play: “ours are normally more … smaller groups I would say” (Peter).

Many participants discussed who they played with or preferred to play with. Most participants preferred to play with other autistic people. This preference was connected to autistic people in a group being similar in some way. For example, Amanda highlighted how play with autistic people uses up fewer resources and is “easier … because we know each other so well, and because we have those similarities in common … it's definitely more relaxed … it's less problematic and it's not as draining as it is if you're with neurotypical people”. In this quotation, Amanda notes the demands of playing with non-autistic people using words such as “draining” and suggests autistic similarities as an explanation for why most participants preferred to play with other autistic people. Similarities include “comparable like, special interests or like sensory things” (Brianna), enjoying “more repetitive things more, silly noises or, you know sensory things or, just sitting nearby while someone else enjoys something rather than … a group activity” (Christine). Here, Christine highlights the previously discussed preference for parallel play.

In contrast, a couple of participants described a preference for playing with non-autistic people. Lauren gave the following justification:

“Everyone says ‘oh yeah you should get on well with this person cos they have autism.’ No actually, no it's not the case, because they have things their way, I have things my way. At least with neurotypical people, the ones that understand you, they accept it and just allow it to happen.”

From this, it seems that playing with other autistic people is less preferred when the group members have different preferences. In this case, play with non-autistic people is preferable provided the non-autistic person is understanding and accommodating, specifically in allowing the autistic person to take the lead. This is reinforced by Ryan who described how play with a non-autistic person is easier “where the neurotypical person knows you have the conditions, [they] will make allowances” but “[an] autistic won't because their objectives are … about themselves in trying to regulate themselves so you … got almost like … two repelling magnets”.

### 2. Imaginary play

We captured experiences relating to imaginary play under two subordinate themes.

#### 2.1. Engaging in imaginary play

Although some participants preferred not to engage in imaginary play, many participants described their engagement in imaginary play and the type of activities they did. Many participants described instances of role-play, especially within a social play context. For example, Andrew described playing the role-playing game Dungeons & Dragons as a “purely social imaginative hobby” and further illustrated how

“I prefer a much more narrative focus to the games. I want it very much to be theatre of the mind, which is a phrase, it's about what you're seeing in your head and how you're seeing your character acting.”

For Andrew, story elements are important to his play, and the use of phrases such as “theatre of the mind” suggests a highly imaginative activity. Lawrence described being part of a historical re-enactment group “where you create a medieval persona and you try and enact that”, while Brianna enjoyed playing with their sister where “we'd pretend to be the characters and we'd like make up our own versions of events that would happen in Lord of the Rings”. Overall, these quotations suggest that enacting the role of a character is an important aspect of these participants' imaginary play.

Not only this, but these quotations highlight that many autistic people do engage in imaginary play. Interestingly, Brianna also reflected on a difference between autistic and non-autistic imaginary play, in that it “looks different”, suggesting that “to me, sitting there lining things up probably doesn't look imaginative at all but while I'm doing that, I've got like this whole thing going on in my brain ((laughs)) that's very imagination based”. This suggests a discrepancy between the observable, which may not appear imaginative to non-autistic people who hold normative assumptions about what imaginary play should be like, and the internal, which is experienced as imaginative.

#### 2.2. Grounded in reality

Many participants (such as Lawrence) preferred imaginary play where the play is grounded in or based on reality. This includes play that is more realistic because it is familiar and based on previous experiences or based more on something potentially real, rather than on fantasy. For example, Lauren described how role-playing teachers “was easier to kind of act out because it's stuff that you've already been through. So you'd always try and like be people that you know, so you can kind of actually act them”.

Similarly, Kevin described how his imaginary play “was something that was based more like off something that I'd seen on the television”. For a couple of participants, the preference for play that is grounded in reality may represent a difference between autistic and non-autistic play. For example, Matthew reflected that:

“Non-autistic children I think have the ability to really use their imaginations. And they can imagine all sorts of things, you know, they get-in terms of pretend games … although I think my experience was that … if we're pretending to do something then, it's sort of, kind of based in reality it's not completely randomly fantastical.”

However, it is worth noting that not all autistic adults may have held this view; some engaged in fantasy play, such as Andrew playing Dungeons & Dragons.

Some participants reflected on whether their preference for grounded-in-reality play counted as imaginary play or to what extent it was imaginary. This is implied by Matthew's description above, but also the following: “I don't know how much of my play was genuinely imaginative and how much of it was actually quite heavily influenced by what I'd seen and what I'd read”. Janet articulated that she “used to be able to do imaginative play and stuff like that, but it used to be quite limited to the things that I was interested in or knew about”. Participants' reflections imply that they perceive that the less imaginative the play is, the more reality-based it is, whereas the more imaginative it is, the more fantasy-based it is. Furthermore, the use of words such as “limited” implies that they perceive their preferred form of play as inferior to less reality-based play.

### 3. Flow

This superordinate theme relates to participants' experiences of a flow state. We captured these under two subordinate themes.

#### 3.1. Experiences of flow

Most participants discussed the idea of a flow state. Participants described their experience of this, which includes two interrelated aspects: time and focus. Most of the participants who related to experiences of flow described the long period of time they spent playing or referred to a sense of time in some way. For example, with respect to video gaming, Kimberly described how she “could literally be on there all day”. In relation to some social play with his friends, Jason described how “there would be a flow state with those as well. We would get really into it, and just keep going for hours and hours and hours and not notice the time passing”. Others had similar experiences, with Lawrence describing how he “could sit for hours upon hours upon hours just, playing” and “can completely lose track of time.”

Relating closely to the time spent playing was the degree of focus involved in participants' play. In the earlier quotation, Jason described how “we would get really into it.” Other participants described being “in the zone” (Brianna), or a “healthy bit of hyper focus” (Fiona), for example. A couple of participants felt that the experience of flow reflected a difference between autistic and non-autistic play. Peter described how play is “generally a lot more intense with us, because we do lose track of time” and Lawrence tellingly pointed out that “somebody not on the spectrum does not get so immersed unless it is football”. Across these accounts, the use of terms such as “immersed” suggests that these participants experience intense focus on their play, as part of their flow experience.

#### 3.2. Benefits and limitations

Many participants described benefits and limitations or difficulties relating to flow. For example, some described mental benefits of flow, including increased relaxation. For example, Matthew described how this experience “took away those stresses, because I was able to wholly concentrate on something which I enjoyed doing”. Here, Matthew points specifically to the intensity of focus as being responsible for the relaxing effect of flow. This is reinforced by Lisa:

“I was so like focused on myself, and what I was doing … yeah it did affect me, but I don't always think it's a bad thing necessarily cos, I think it eases like tension for me not having to notice much.”

Interesting here is Lisa's awareness of the potential downsides of flow, as well as its benefits. Like Lisa, many participants talked about *both* benefits and limitations of flow. For instance, Karen showed her awareness of both by describing how flow is “comfortable up to a point”.

Many participants described limitations around the negative impact of flow on aspects of everyday living, particularly self-care. For Lisa, the downside of flow is that she does not “always have the best sleep schedule”. Janet echoed this impact on sleep while also describing effects on eating and not doing other important activities: “I can lose lots of time, engaging in that social play and not getting actually what I need to do done, or not going and eating or not going and sleeping”.

## Discussion

Through our neurodiversity-informed study, in which we centered autistic perspectives and adopted a balanced rather than a deficit-focused interpretive lens, we generated some important insights into autistic adults' perspectives on how they construct their play experiences. We investigated what is important about their play, and how autistic play is different from non-autistic play. We found important commonalities and differences in the ways that socialization in play, imaginary play, and flow are experienced.

### Socialization in play

We found that both solitary play and social play are important for many autistic adults and that these types of play have different purposes: solitary play for relaxing or recuperating and social play for social benefits and skill development. Interestingly, some autistic adults' preference depended on their resources or needs at a specific moment, with solitary play preferred when they were tired or had limited resources. This seemed linked to the demands of social play, which was described as a “double-edged sword” because of its benefits and drawbacks. The tiring nature of social play echoes what is found in the literature on navigating friendships and socializing more generally.^43–46^

Our findings suggest that autistic adults' preferences with respect to these types of play are not necessarily fixed. These findings contrast with the typical view that autistic people have social play “deficits” and are consistent with neurodiversity-informed play literature suggesting that autistic people do engage in social play.^29–31,33^ Our finding of social play benefits is consistent with Pavlopoulou et al.'s^33^ research, particularly in relation to play facilitating a sense of belonging. Our study additionally builds on previous research by highlighting the specific benefits of solitary play (relaxing or recuperating) and indicating why some autistic adults may prefer solitary play or social play at a given moment.

Many autistic adults discussed their preferences in social play, which relate to social play differences and who they preferred to play with. We found some potential social play differences, such as some preferring smaller groups and some autistic adults describing autistic social play as similar to parallel play. More specifically, this is characterized by close proximity and no requirement for interaction (although this may take place). This fits most closely with Parten's^9^ concept of parallel play, although we recognize the tensions with applying non-autistic definitions to describe autistic experiences: it is important to base the definition of parallel play for this study on autistic people's views.

This finding corroborates Fahy et al.'s^31^ study in which autistic children were often observed to play in close proximity, although in their study the children's social play took on different forms, and this did not necessarily involve parallel play. This also echoes previous research in which parallel play was observed as one of the most frequent play states for autistic children in free play settings.^47^ Nonetheless, the findings of the current study highlight that for some, autistic social play is like parallel play, and it is important to view this as a social play preference that is different from but not “less” than forms of social play that are usually viewed as more interactive and superior by non-autistic people.^8,9^

We also found that most autistic adults preferred to play with other autistic people, potentially because of similarities between autistic group members, such as having similar play preferences. The findings are consistent with Fahy et al.'s^31^ finding that autistic children seemed to prefer playing with their autistic peers compared with a mainstream class and support their argument that this might be due to their play similarities.^29^ The various similarities highlighted by autistic adults suggest that autistic–autistic similarities in interacting and communicating operate in a social play context and support the idea that autistic social play may be different from non-autistic social play. More broadly, our findings are consistent with wider research arguing that autistic communication is different and that autistic people tend to find it easier and less tiring interacting with other autistic people. As most autistic adults in our study preferred to play with autistic rather than non-autistic people, our findings are consistent with the double empathy problem.^41,45,48^

It is important to note that a minority preferred to play with non-autistic people. This occurred under specific circumstances, where autistic group members' preferences differed and non-autistic people are understanding and accommodating, specifically in allowing the autistic person to take the lead. These findings are consistent with previous research highlighting positive play experiences with non-autistic people who are sensitive to autistic people's preferences and accommodate these within their social play.^29,30^

Overall, the findings suggest that autistic people prefer to play with other autistic people as long as they are similar in some way, or non-autistic people who are supportive of accommodating autistic people's play preferences. These findings highlight the importance of supporting autistic people to play with each other and of non-autistic people accommodating autistic preferences.^45^ For example, autistic-friendly environments could be promoted in various contexts (e.g., education) in which autistic people take the lead. However, consideration should be given to individual preferences and needs regarding play partners.

### Imaginary play

Many autistic adults in this study described their engagement in imaginary play, particularly relating to social role-play. This supports other neurodiversity-informed literature finding that autistic people do engage in imaginary play, and contrast with deficit-focused views that autistic imaginary play is limited.^29–31,33^ More specifically, our findings add to the existing evidence base on autistic engagement in role-play in various forms, such as gaming and live action.^33,49–52^

We also gained insight into the kind of imaginary play that autistic adults preferred, with many preferring play that is grounded in reality, such as play based on something previously experienced (e.g., from a television show), or is realistic. This is in line with some research^29,30^ where some participants preferred or engaged with imaginary play that is close to or recreates experiences from real life. One possible reason for this may be autistic people's preference for familiarity and predictability. Combined with our finding that a couple of participants viewed this preference as a difference between autistic and non-autistic play, this suggests that autistic imaginary play may differ from non-autistic play in this respect.

However, this finding contrasts with previous studies showing autistic engagement or preference for fantasy.^33,51,52^ Indeed, some autistic adults in the current study engaged in fantasy play (e.g., Dungeons & Dragons). This further highlights the wide variation in autistic people's play preferences and is reminiscent of research with non-autistic children showing that individuals vary in fantasy orientation (e.g., Weisberg^53^). Overall, it is important to acknowledge individual differences with respect to autistic preferences for fantasy- or reality-based play.

### Flow

Most autistic adults in this study experienced a flow state in relation to their play, involving intense focus on a play activity for a long time and possibly an altered sense of the passage of time. Intense focus is the defining characteristic of flow,^32^ and our findings, combined with previous research,^31,33^ suggest that this is also a defining characteristic of autistic play flow experiences. More broadly, the combination of previous research support and our finding that a couple of participants viewed the experience of flow as a difference between autistic and non-autistic play suggests that autistic engagement in flow is a potential characteristic of at least some autistic people's play. As highlighted by previous research, autistic people's engagement in flow may be a consequence of monotropism,^33,34,54^ where autistic people's intensely focused attention on few interests or activities means immersion in an activity and entering into a flow state is more likely. As play may be a form of interest, it is therefore unsurprising that many autistic people experience flow during play.

We also found that many experienced both benefits and limitations of flow, which suggests that for autistic people, flow has a dual nature. Our finding of mental benefits of flow, including relaxation, suggests that this experience could be important for autistic people's well-being.^32,34^ However, autistic adults also discussed the negative impact of flow on aspects of everyday life, particularly self-care, which others have highlighted.^33,34^ Our findings parallel those of Pavlopoulou et al.,^33^ particularly in relation to the negative impact of flow on sleeping and eating. We build on this research by also highlighting relaxation as a specific benefit of flow. Overall, the findings suggest that it is important to acknowledge the dual nature of flow relating to autistic people's play experiences.

### Strengths and limitations

Our neurodiversity-informed approach, both methodologically and through the interpretative lens we adopted, is a notable strength of this study. Another strength of our study was the community involvement, particularly of autistic people, which informed the topic of the research and its methodology. However, we would have benefited from greater autistic involvement, particularly in the analysis stage. Strategies such as credibility or member checking would ensure that interpretations reflect as closely as possible participants' meanings.^39,40^ However, due to the COVID-19 pandemic disrupting the timeline and research operational capacity, we were unable to carry out member checking. We kept participants updated on research progress by email. Furthermore, adopting approaches beyond consultation, such as co-produced or autistic-led research, would give us better and more meaningful understandings of autistic play.^26^

Another limitation is that our sample consists predominantly of autistic adults who are White, highly educated, and primarily communicate using speech. Although the goal of qualitative research is not generalizability, future research would benefit from exploring autistic play in underrepresented groups to capture a diversity of perspectives. Additionally, although our sample consisted of a balance of adults who identified as male and female, greater inclusion of autistic people who identify as nonbinary or transgender (or other gender identities) would be beneficial.^55^

### Implications and conclusions

Supporting the importance of taking an individual approach to play, we underline some of the different ways some autistic adults experience play. We highlight the importance of both solitary play and social play and the different circumstances under which each of these is preferred. We also highlight social play preferences such as parallel play and playing with similar autistic people, imaginary play experiences including social role-play and grounded-in-reality play, and the dual nature of flow experiences during play. Overall, the findings of this study contrast with deficit-focused understandings of autistic play and build on the small evidence base providing neurodiversity-informed understandings. Importantly, we have done so by centering autistic perspectives and interpreting the data from a balanced rather than deficit-focused perspective.

These findings have important implications for future research. As the evidence base in this area is in its early stages, and IPA does not seek to make generalizations, it is important to explore whether other autistic people experience play in the ways we highlighted. Furthermore, future research would benefit from exploring specific areas in more depth. This includes the characteristics of autistic flow states and how these relate to monotropism, autistic conceptualizations of social play (e.g., “parallel” play), and how autistic play supports autistic people's well-being.

Furthermore, our findings have implications for autism diagnostic assessment, where play often forms a key component, for example, as part of the *Autism Diagnostic Observation Schedule*.^56,57^ Our findings suggest play preferences that could be included in diagnostic assessment practices outside of a deficit-focused model, for example, by including observation of or discussions about grounded-in-reality play, self-regulation through solitary play, and engagement in flow. Further research is required to explore and validate these possibilities.

Our findings also have implications for settings involving potential social play (e.g., education, recreation). For example, it is important to provide environments where autistic people can play solitarily should they choose to do so.

More generally, we encourage professionals, researchers, and members of wider society to become more understanding, accepting, and supportive of the various play preferences and experiences we have highlighted, and to avoid using deficit-focused language and mindsets in relation to autistic play. We believe that supporting authentic autistic play could reduce factors that lead to stigma and masking^24,25^ and therefore contribute to positive mental health and well-being for autistic people.

## Supplementary Material

Supplementary Material 1

Supplementary Material 2
