# Supplementary material for: Diversity in Autistic Play: Autistic Adults' Experiences
Source: Autism Adulthood. 2024 Jun 17;6(2):218–28. doi: 10.1089/aut.2023.0008 (PMC11317800; doi:10.1089/aut.2023.0008)
Supplement: Supplementary Material 1 [file aut.2023.0008_suppl_data1.pdf]

CONVERSATION TOPIC GUIDE

Play & Communication Lab
making connections through play * paclab.co.uk
PI: Dr Jenny Gibson

This detailed topic guide includes the interview schedule used by the research assistant in the interview.
It outlines the topics we will cover in order and some questions and prompts that will be asked and used under each topic.
This is a semi-structured interview and we do not plan to ask each participant every single question or prompt listed.

**Introduction**	Discussion of environment	Are you happy with how you can see me and hear me?<br><br>Is there anything that would be helpful for me to know that I can adjust or do to make the interview a positive experience?	
	About the study	This study is looking at the experience of autistic play from an autistic perspective.<br><br>I am having this interview with you because I want to understand your experience of play. We will be looking for themes and patterns which come up across both yours other participants interviews.	
	Information sharing	I want to also confirm that you understand that if you share some information which I feel might mean you or someone else could be harmed, I would stop the interview. I would discuss this with you and ask what you would like to do next.<br><br>I would tell you if I felt I have to share that information with someone else.	
	Informed Consent	I know you have already read the information sheet and signed the consent sheet.<br><br>I want to check you know this interview will be recorded, and then later written out word for word. This will be kept separate from your personal information such as your name, so you won't be identified. Do you have any questions?	
		I think our interview will be around 20-40 minutes but we can talk for as little or long as you would like up to a limit of one hour. I have suggested a 5 minute break halfway through and you can also request more.	

		Are you happy to get started? We can stop at any time and you do not have to answer any questions which you are not comfortable with.	

Supportive prompts used throughout<br><br>Can you give me a bit more detail.<br><br>Can you tell me more.<br><br>Clarification of points by summarising.

**Your experiences of play**	Tell me about your favourite activity or interest.	Prompt questions if relevant:<br><br>How long has this been your favourite thing to do?<br><br>When do you do it?<br><br>How often do you do it?<br><br>How does it make you feel?<br><br>Would you consider this play?	Example prompts<br><br>Use supportive prompt first<br><br>‘I like to xxxx at the weekend, what about you?’<br><br>‘On the weekend I spend my time ….’
	What would you tell someone who had never played with an autistic person about your play? This could be your play now or when you were younger.	Prompt questions if relevant:<br><br>What kind of activities were involved?<br><br>What kind of play experiences did you enjoy growing up and even now?<br><br>What do you consider to be play / games?<br><br>Is there a process or structure you follow?<br><br>How would your play be affected by who you were with or where you were?<br><br>How does it make you feel?<br><br>What made you feel good/engaged?<br><br>Can you give me an example of a play experience which has gone really well?<br><br>Can you give me an example of one which hasn’t gone so well?<br><br>Do you think there are stereotypes in autistic play and do you think these are accurate? What would you say they are?	Example prompts<br><br>Use supportive prompt first<br><br>For example, you could talk about what you would play at home when you were younger.<br><br>“When I was younger I often played…”

		Do stimming, repetitive activities or intense interests (change these examples depending on which stereotypes were given) have a role in your play?<br><br>What allows you to play how you want to?	
	Is play beneficial to you?	Does it ever have a purpose or a benefit to you? (For example other autistic people we have talked to have mentioned a reduction in anxiety, a feeling of connectedness/sensory feedback/calming)<br><br>It helps some people to cope - is that true for you?<br>For example with learning, life, friendships etc?<br><br>Could you tell me why?<br><br>Are there different kinds of activities that you would engage with at different times?<br><br>Can it also have a negative effect?	Example prompts<br><br>Use supportive prompt first<br><br>I think really active play when I was younger helped me to stay relaxed and feel free. I used to play running games on the street with my neighbours and I enjoyed the adrenalin rush from that.<br><br>"Playing would make me feel….."
	Sometimes when I play, I get the balance right between the challenge in an activity and my own skill level.<br><br>Example of art/music and forgetting time passing/eating.<br><br>Sometimes I feel the complete opposite and get bored if the piece of music is too easy, and maybe frustrated and/or anxious if it's too hard.<br><br>How do these ideas relate to your own play experiences?	Are there activities that you find you can do for hours without noticing the time?<br><br>Are there any types of play that you find frustrating?	Example prompts<br><br>Use supportive prompt first<br><br>"I forget the time and can spend hours doing…."<br><br>"one activity I can only do for a very short amount of time is…"

	Sometimes people talk about play as a way to push boundaries and take risks. This could relate to the physical, the virtual or the social world. Do you relate to this?	Do you do adventurous things - that push physical boundaries<br><br>Do you enjoy pretending to be someone else or live in a different way to normal?	Example prompts<br><br>Use supportive prompt first<br><br>For example adrenalin provoking or stimulation seeking activities, role play or cos play.<br><br>Some people enjoy cos play as they like to become a different character.<br><br>I myself have pushed my adrenalin boundaries through sky diving and scuba diving and other people enjoy playing computer games where they get to play characters who behave violently.<br><br>"The ways in which I push boundaries in play are..."

{BREAK HERE}

**Comparing your play to neurotypical play**	Thinking about your description of your play, how does it compare to other people's play	Prompt questions if necessary:<br><br>How important is it to you, that you get to choose the activities you take part in, rather than someone else choosing the activity?<br><br>Does it help you to connect with people and build relationships? Can you compare this to what you see in other people's play?<br><br>What do you enjoy?<br>What kind of play keeps your attention?<br><br>Do you think you have to be visibly engaged and happy/joyful to be playing?<br><br>Are there other ways that you feel autistic play is similar/different to other types of play?	Example prompts<br><br>Use supportive prompt first<br><br>For example, other people in your friendship group, other people who are not autistic.

		How do you feel playing with other autistic people compared to other neurotypical people?	
	Do you feel that autistic play is fully understood in society in general?<br><br>What would you like more people to know about autistic play?	Do you get to play as much as you would like to/need to?<br><br>What are some common misconceptions about autistic play?<br><br>What would make it easier for you to play in the way you enjoy most? What about as a child/teenager?	<u>Example prompts</u><br><br>Use supportive prompt first<br><br>I personally think that autistic play is not understood, and that I would like to see more understanding from others.<br><br>“I want people to understand that…”
	Sometimes people make a distinction between solitary play and social play. Tell me about your experiences regarding this?	Do you enjoy one type more than the other?<br><br>What makes for the best social play experiences?<br><br>What makes for the best solitary play experiences?<br><br>Are there different ways of engaging in social play in your view? Anything that you see as specific to autistic play?<br><br>How do shared or common interests affect your engagement and enjoyment of social play?<br><br>Do you think that social play is more enjoyable with other autistic people or neurotypical people, or does that not effect it?	<u>Example prompts</u><br><br>Use supportive prompt first<br><br>For example, a young child playing alone in the sandpit would be solitary play or playing cops and robbers with friends would be social play.

{OPTIONAL BREAK HERE}

**Experience of assessment and intervention using play**	Sometimes play and games are used as part of therapy, support or assessment for autistic people. Can you tell me about your experiences with this?	<u>Prompt questions if necessary:</u><br><br>Have you received a report about yourself which discusses the way you play?<br>What did you think about it? Was there a good balance between strengths and challenges?<br>Was anything missing?<br><br>In some autism assessments, for example ADOS, participants are asked to act out a ‘play’ or pretence scenario - what was your experience of this?	<u>Example prompts</u><br><br>Use supportive prompt first<br><br>My experience with this is in support rather than assessment. One example is supporting very young autistic children – where we would play with toys in a very guided way and I

		What are your experiences of play being used as a tool for social support or intervention? Do your views change depending on the type of support offered - eg social skills group, peer mentoring, therapeutic video games<br><br>What advice would you give to a professional who is using play as part of an intervention or support?	used simple language to help them develop their language skills.<br><br>“When I was diagnosed with autism I played with….”<br><br>“In school I received support from….”
	Sometimes differences in the way autistic people play are used as part of autism diagnostic assessments. In your opinion, what would these differences be for you?	How would these differences best be identified by a professional who is deciding whether to give you a diagnosis	<u>Example prompts</u><br><br>Use supportive prompts first<br><br>For example, younger autistic children might spend a long time exploring an object and investigating its sensory properties<br><br>“The differences in my play that would be identified in an autism diagnosis assessment are…”<br><br>For example, differences in play could be looked at by taking part in a free play activity with you, through a guided or structured play activity, through a discussion or by asking you.<br><br>“A professional could identify the differences in my play by…”
**Summary**	We have reached the end of the interview.<br>Is there anything else you would like to share?<br><br>Would you like to reflect on this interview experience - for example- was it easy to talk about your play?		(Give some time - couple of minutes reflection)

	If you think of anything else you would like to share please feel free to email it to me.<br><br>I will send you a thank you email after this interview.<br><br>Once we have thought about this conversations, we will send you our thoughts on it for you to comment on.		
