# Supplementary material for: Diversity in Autistic Play: Autistic Adults' Experiences
Source: Autism Adulthood. 2024 Jun 17;6(2):218–28. doi: 10.1089/aut.2023.0008 (PMC11317800; doi:10.1089/aut.2023.0008)
Supplement: Supplementary Material 2 [file aut.2023.0008_suppl_data2.pdf]

Supplementary Material 2: Positionality statement

The first author is a researcher holding degrees relating to (Clinical) Psychology. She seeks to counter deficit-based understandings of autistic ways of being through conducting research from a strengths-based or neurodiversity-informed perspective. This informed her approach to interpreting the data in this study.

The second author is a non-autistic speech and language therapist, specialising in autism. At the time of the study design, community involvement and participant interviews, the author was working as a research assistant and was interested in supporting the development of a strengths-based research strategy for the research centre. The author is currently employed in clinical work supporting an inner-city primary-aged paediatric client base.

The third author is a non-autistic researcher, with particular interests in autistic experiences, the neurodiversity paradigm, mental health and the use of interpretative phenomenological analysis.

The last author is a research psychologist and speech and language therapist. Personal and family lived experience as well as academic specialism in neurodivergent play have informed her approach to research design and interpretation.
